# Biologics-associated Lichen Striatus-like eruption: insights from two case reports and literature review^؆^

**DOI:** 10.1016/j.abd.2026.501405

**Published:** 2026-06-20

**Authors:** Liang Zhao, Yaoying Gao, Liyun Dong, Nuoya Zhou, Yamin Zhang, Jing Yang

**Affiliations:** Department of Dermatology, Affiliated Union Hospital, Tongji Medical College, Huazhong University of Science and Technology, Wuhan, China

Dear Editor,

Lichen striatus (LS) is a linear dermatitis of unknown etiology, characterized by unilateral papules on Blaschko's line.^1^ Although associations with trauma, viral infections, and genetic predisposition have been proposed, the pathogenesis remains unclear.^2^ With the expanding use of biologics in immune-mediated diseases.^3^, ^4^ We herein present two cases of lichen striatus-like eruption emerging during biologic therapy.

## Case 1

**Fig. 1:**
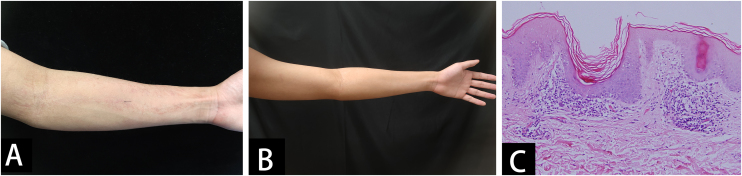
Lichen striatus -like lesions developed following IL-4R inhibitor treatment. (A) Clinical manifestations of the AD patient after 4-months dupilumab treatment, and (B) Lichen striatus -like eruptions resolved after 4-weeks therapy. (C) the histopathological examinations of the lichen striatus -like lesions of the AD patient (Hematoxylin & eosin, ×200).

A 20-year-old male with severe atopic dermatitis (AD) for over 10-years achieved complete clearance within 3-months of initiating IL-4/IL-13 pathway inhibitor (dupilumab). Four months later, asymptomatic erythematous papules appeared on the left forearm (Fig. 1A). Histopathological examination revealed mild hyperkeratosis with necrotic keratinocytes; acanthosis and spongiosis. Infiltration of lymphocytes and histiocytes was observed in the upper dermis and around the vascular regions (Fig. 1C). There was no mucosal or nail involvement. The patient had no recent infection history and denied taking any medications other than dupilumab. With topical halometasone, lesions resolved in four weeks with post-inflammatory hypopigmentation (Fig. 1B). Dupilumab was maintained for 24 additional months without the lichen striatus-like eruption recurrence.

## Case 2

**Fig. 2:**
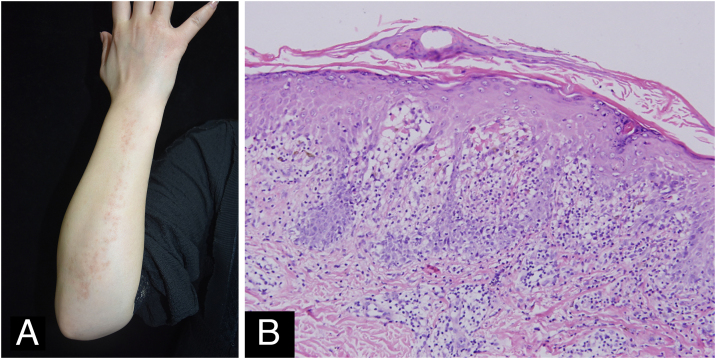
Lichen striatus -like lesions developed following IL-23 inhibitor treatment. (A) Clinical manifestations and (B) Histopathological examinations lichen striatus -like lesions of the psoriasis patient after 4-months guselkumab treatment (Hematoxylin & eosin, ×100).

A 32-year-old female with plaque psoriasis achieved complete remission after starting an IL-23 inhibitor (guselkumab). Four months later, linear erythematous papules developed on the right forearm (Fig. 2A). Dermoscopy showed erythematous papules on a reddish background without Wickham's striae. Histopathology features were similar to those of Case 1 (Fig. 2B). The final diagnosis was lichen striatus-like eruptions based on the clinical presentation and histopathological examinations. The lesions improved within 6-weeks using halometasone cream, with no recurrence over 24-months of continued guselkumab therapy.

## Discussion

Linear lichenoid eruptions is a rare papulosquamous disorder in adults. To our knowledge, these cases represent the first reports of such lichen striatus-like eruptions associated with IL-4/IL-13 or IL-23 inhibition. While lichen striatus has previously been linked to TNF-α inhibitors (etanercept) and IL-17 blockade (secukinumab),^5^, ^6^ our findings expand the spectrum of biologic-associated paradoxical cutaneous reactions.

The attribution of the lichen striatus-like eruption to the biologic agent, while temporally correlated, warrants careful consideration. According to the Naranjo criteria for adverse drug reactions,^7^ a definitive causal relationship is supported by resolution upon drug discontinuation, which was not performed in our cases, as the biologics were deemed essential for controlling the severe primary disease. The improvement observed could be attributed to the natural course of eruption or the effect of the topical corticosteroid therapy. Nevertheless, multiple factors support a plausible association with biologic therapy. First, the onset occurred within a typical window (4-months) for paradoxical reactions to biologics. Second, lichen striatus-like dermatitis is rare in adults, making its appearance in two patients on effective biologic therapy notable. Finally, existing literature indicates that a substantial proportion of biologic-associated cutaneous reactions resolve spontaneously without necessitating drug withdrawal, a pattern consistent with our cases^8^ (Table 1).^5^, ^6^ Although a definitive causal link cannot be established, the presented evidence suggests a probable association worthy of clinical awareness and further investigation.

**Table 1:** Reported Lichen Striatus-like lesions after biologics treatment.^5^, ^6^.

Study	Patient age, y/sex	Underlying disease	Drug	Reaction	Clinical morphology	Time to reaction	Cessation of biologics	Outcome
Viviana Lora et al. 2014^5^	70/F	Rheumatoid arthritis	Etanercept	Leg	Erythematous, scaly, papular	8-months	Yes	Recovery
C.‐W. Yang et al. 2017^6^	33/M	Psoriasis	Secukinumab	Thigh	Hypopigmented macules	8-weeks	No	Improvement
Current case study 1	20/M	Atopic dermatitis	Dupilumab	Forearm	Erythematous, papular	4-months	No	Recovery
Current case study 2	32/F	Psoriasis	Guselkumab	Forearm	Erythematous, papular	4-months	No	Improvement

The precise classification also presents a challenge, primarily between lichen striatus (LS) and linear lichen planus (LP). Clinically, our cases favor LS (asymptomatic, erythematou papules on Blaschko's line without Wickham's striae or pruritus). Histopathologically, however, features were equivocal: focal basal vacuolization and a lichenoid interface dermatitis suggest LP, yet the absence of a dense band-like infiltrate and the presence of only mild spongiosis deviate from classic LP and LS, respectively. This hybrid profile justifies the descriptive designation “lichen striatus-like dermatitis” and underscores that inflammatory dermatoses arising in the context of novel biologics may not conform to classic histopathological archetypes.

The pathogenesis of such reactions may involve cytokine network disruption. LS is characterized by epidermal CD8^+^ T-cell infiltration. We hypothesize that dupilumab, by inhibiting IL-4/IL-13 signaling, may shift immunity toward Th1/Th17 polarization, elevating IFN-γ/CXCL9-10 and recruiting CD8^+^ T-cells to keratinocytes. Guselkumab, through IL-23 blockade, could attenuate IL-17-mediated suppression of CD8^+^ T-cell pathways. This hypothesis is supported by studies on TNF-α inhibitors, which have shown IFN-α-driven inflammation resulting from disrupted cytokine networks.^9^

In summary, these cases suggest lichen striatus-like dermatitis may represent a paradoxical reaction to IL-4/IL-13 and IL-23 inhibitors. Clinicians should consider this entity in patients developing eruptions on Blaschko's line during biologic therapy. Further research should characterize these linear lichenoid eruptions-associated lymphocyte subsets and cytokine profiles to establish mechanistic links to biologic therapies.

## ORCID ID

Liang Zhao: 0000-0002-5796-0913

Yaoying Gao: 0009-0004-6803-2284

Liyun Dong: 0000-0001-5377-0752

Nuoya Zhou: 0000-0002-0823-8844

Yamin Zhang: 0000-0002-4170-5897

## Financial support

This work was supported by the National Natural Science Foundation of China (grant number 82473527).

## Authors' contributions

Liang Zhao: Study conception and planning; preparation and writing of the manuscript; manuscript critical review; approval of the final version of the manuscript.

Jing Yang: Study conception and planning; data collection, analysis and interpretation; preparation and writing of the manuscript; manuscript critical review; approval of the final version of the manuscript.

Yaoying Gao: Data collection, analysis and interpretation; approval of the final version of the manuscript.

Liyun Dong: Data collection, analysis and interpretation; approval of the final version of the manuscript.

Nuoya Zhou: Data collection, analysis and interpretation; approval of the final version of the manuscript.

Yamin Zhang: Data collection, analysis and interpretation; approval of the final version of the manuscript.

## Research data availability

Does not apply.

## Conflicts of interest

None declared.
